# Subjective Outcome Evaluation of the Project P.A.T.H.S: Descriptive Profiles and Correlates

**DOI:** 10.1100/tsw.2010.23

**Published:** 2010-02-12

**Authors:** Daniel T.L. Shek, Lu Yu

**Affiliations:** ^1^ Department of Applied Social Sciences, Hong Kong Polytechnic University, Hong Kong; ^2^ Public Policy Research Institute, Hong Kong Polytechnic University, Hong Kong; ^3^ Kiang Wu Nursing College of Macau, Macao

**Keywords:** positive youth development program, Hong Kong, subject outcome evaluation, Chinese adolescents

## Abstract

A total of 196 schools participated in the Tier 1 Program (Secondary 2 curriculum) of the Project P.A.T.H.S. (Positive Adolescent Training through Holistic Social Programmes) in Hong Kong. After the completion of the program, 1,178 instructors completed a subjective outcome evaluation form in order to assess their views of the program, instructors, and perceived effectiveness of the program. Results showed that high proportions of the instructors had positive perceptions of the program (range: 76.79–93.69%) and their own performance (range: 83.20–98.60%), and most of the respondents (range: 78.45–92.43%) regarded the program as helpful to the program participants. While the ratings in some items in the present findings were relatively better than those in the Experimental Implementation Phase, they were similar to those based on the Secondary 1 curriculum. Consistent with previous studies, the ratings on the program, instructors, and perceived effectiveness were significantly correlated.

### INTRODUCTION

The Project P.A.T.H.S. (**P**ositive **A**dolescent **T**raining through **H**olistic **S**ocial Programmes) is a two-tier positive youth development program. The Tier 1 Program is a universal positive youth development program, typically involving 20 h of training (40 units) at each grade for Secondary 1 to 3 students in each school year. The Tier 2 Program aims to help students with greater psychosocial needs, such as those lacking psychosocial competencies[1]. The Project has two phases: from the school year 2005/06 to 2007/08 was the Experimental Implementation Phase, and starting from the school year 2006/07 was the Full Implementation Phase. As a positive youth development project funded by The Hong Kong Jockey Club Charities Trust, the Project P.A.T.H.S. can be regarded as an exemplary program that systematically utilizes research findings in order to understand adolescent developmental issues, develop a positive youth development program, and evaluate the developed program[2].

For program evaluation, Denzin[3] used the term “triangulation” to argue for the utilization of different types of data based on different methodologies to examine the same phenomenon. When different methods, data, and/or investigations are involved, biases and errors that exist in any single type of investigation can be revealed and cancelled out. Actually, this is a commonly adopted approach in program evaluation in the postpositivistic paradigm. In the Project P.A.T.H.S., triangulation by data sources (e.g., views of both program implementers and participants about the effectiveness of the program), triangulation by different methods (objective outcome evaluation, subjective outcome evaluation, evaluation based on repertory grid tests, and process evaluation), triangulation by researchers (inter-rater reliability checking), and triangulation by data types (quantitative data and qualitative data) are carried out[2,4,5].

To evaluate the Tier 1 Program, Shek et al.[6] suggested that in addition to understanding the program participants’ experiences, it is equally important to examine the perceptions of the workers who implement the program. Involving the implementers’ perceptions in the evaluation would offer more accurate views, promote reflective practice in the profession, nurture a sense of fairness and respect, provide opportunities for rumor demystification, and triangulate the results. Reppucci[7] also indicated that intervention programs developed by researchers in specially funded or university-based situations may not be well implemented by social workers or clinicians who usually are required to implement the program in the context of a complex array of sociopolitical realities. Since school administrators and teachers are the “primary adopters” of such programs, their support is essential for the continuation of prevention programs within the school setting. As shown in the research of Flannery and Torquati[8], “teachers who are not satisfied with a program are less likely to use the program materials, regardless of whether their principal or district administration is supportive of the program” (p. 395). Furthermore, an increasing number of researchers have recently advocated that program evaluation should not only assess the merit of a program’s past performance, but also lead to the development of lessons that will help program staff to improve program implementation in the future[9,10,11]. Obviously, the program staff has a particular role to play in providing their observations regarding the activities being implemented and their suggestions on how the program can be improved. Based upon the views of program implementers, program managers/researchers can make better decisions about how to adjust the program strategies and activities. Hence, in evaluating as well as monitoring the implementation of a school-based Tier 1 Program, the views of the program implementers must be taken into account.

Subjective outcome evaluation is one way to assess the program implementers’ satisfaction with the program and their perceived changes in the participants. Client satisfaction surveys are frequently used as feedback for transforming services to meet the users’ needs for further planning and administration purposes, and also used as an indicator of program effectiveness from the participants’ or implementers’ perspectives for research purposes. Several studies[12,13,14] utilizing subjective outcome evaluation have documented the program implementers’ positive views and perceived benefits toward the Tier 1 Program, which provide support for the effectiveness of the P.A.T.H.S. Project. Generally speaking, the findings suggest that both the program participants and program implementers perceived the program in a positive manner.

However, existing findings about implementers’ perceptions on the Secondary 2 (S2) program are limited to 52 schools in the Experimental Implementation Phase[12,13]. Although similar results have been obtained from instructors who implemented the Secondary 1 (S1) program[14] in the Full Implementation Phase, such positive findings may not be generalized to the S2 level. Several reasons necessitate further investigation of the program implementers’ subjective evaluation about the S2 program. First, S1 students differ from S2 students in several aspects, such as their psychological maturity and developmental needs. Researchers have long suggested that with augmented cognitive complexity and life experience, adolescents’ prosocial and moral behavior increases as they get older[15]. In a similar vein, Saarni[16] summarized that from age 13 (i.e., S2 level) onwards, adolescents become more aware of their own emotions and those of others, and are capable of coping with stress more effectively[17]. S2 students also show more adjustment problems as compared to the S1 students[18]. Second, S2 students have already attended the S1 program in the preceding year. These students will be more familiar with the

Project P.A.T.H.S. and have more or less gained from this program. The implication is that S2 students may be more actively involved in the program than S1 students, and thus the instructors would perceive the S2 program as more effective. Of course, it can be counterargued that with previous exposure to the S1 program, students may feel bored and uninterested, which may eventually affect the program effectiveness.

Third, the content of the S2 curriculum is somewhat different from the S1 curriculum. For example, for the same purpose of fostering good communication and bonding, while the S1 curriculum emphasizes the promotion of trust and understanding among peers and teachers, the S2 curriculum focuses on promoting bonding with parents in the family context[19]. Fourth, in the second year of the Full Implementation Phase, both instructors and other related personnel (e.g., principals or senior administrators) will have a better understanding of the project and have accumulated a wealth of experience in implementing the program. As such, the implementers' perceptions about the program may have changed accordingly.

Against the above background, it is important to understand how the program implementers evaluate the S2 program in the Full Implementation Phase. Besides presenting descriptive profiles about the responses of the program implementers, the present study also examined three other issues. First, we compared program implementers' evaluations about the S2 program in the Full Implementation Phase with those in the Experimental Implementation Phase. With more practice and experience, it was expected that program implementation would be better in the Full Implementation Phase than in the Experimental Implementation Phase. Therefore, we hypothesized that the instructors' perceptions about the S2 program in the Full Implementation Phase would be better than in the Experimental Implementation Phase. Second, we examined differences between program implementers' evaluations about the S2 and S1 program in the Full Implementation Phase. Because of the complexity of this issue as discussed in the preceding paragraphs, no specific hypothesis was put forth. Third, the question of how various aspects of implementers' evaluation about the S2 program may be intercorrelated was tested. According to previous research findings[5,13], it was predicted that ratings on the program, instructors, and perceived benefits would be significantly correlated.

### METHODS

#### Participants

There were 196 schools that joined the Secondary 2 program of the Project P.A.T.H.S. in the second year of the Full Implementation Phase in the school year 2007/08. The mean number of students per school was 170.66 (range: 12–280), with an average of 4.63 classes per school (range: 1–8). Among the 196 schools, 83 schools adopted the full program (i.e., 20-h program involving 40 units), whereas 113 schools adopted the core program (i.e., 10-h program involving 20 units). The mean number of sessions used to implement the program was 22.81 (range: 7–60). While 108 (55.10%) schools incorporated the program into the formal curriculum (e.g., Liberal Studies, Life Education), 88 schools (44.90%) used other modes (e.g., using form teachers' periods and other combinations) to implement the program. A total of 1,486 workers implemented the program in the schools, with an average of 1.97 social workers (range: 0–8) and 5.59 teachers (range: 0–15) per school.

After the Tier 1 Program was completed, all the implementers (both social workers and teachers) were invited to respond to a subjective outcome evaluation questionnaire. A total of 1,178 implementers completed the Subjective Outcome Evaluation Form (Form B) developed by the research team. The data collection was normally carried out after the completion of the Tier 1 Program. To facilitate the program evaluation, the research team developed an evaluation manual with standardized instructions for collecting the subjective outcome evaluation data[20]. In addition, adequate training was provided to implementers during 20-h training workshops on how to collect and analyze the data collected with Form B.

### Instruments

The Subjective Outcome Evaluation Form (Form B) was designed by Shek et al.[20]. Broadly speaking, there are 10 parts in this evaluation form, as follow:

1. Program implementers' perceptions of the program, such as program objectives, design, classroom atmosphere, interaction among the students, and the respondents' participation during class (10 items).
2. Program implementers' perceptions of their own practice, including their understanding of the course, teaching skill, professional attitude, involvement, and interaction with the students (10 items).
3. Workers' perceptions of the effectiveness of the program on students, such as promotion of different psychosocial competencies, resilience, and overall personal development (16 items).
4. The extent to which the workers would recommend the program to other students with similar needs (one item).
5. The extent to which the workers would teach similar programs in the future (one item).
6. The extent to which the program implementation has helped the workers' professional growth (one item).
7. Things that the workers obtained from the program (open-ended question).
8. Things that the workers appreciated most (open-ended question).
9. Difficulties encountered (open-ended question).
10. Areas that require improvement (open-ended question).

For the quantitative data, the implementers who collected the data were requested to input the data into an EXCEL file developed by the research team that automatically computes the frequencies and percentages associated with the different ratings for an item. The schools were required to submit both hard and soft copies of the consolidated datasheets. After receiving the consolidated data by the funding body, the research team "reconstructed" an overall profile of the subjective outcome evaluation by aggregating the data.

## RESULTS

The quantitative findings based on the closed-ended questions are presented in this paper. Reliability analysis with school as the unit of analyses showed that Form B was internally consistent: for the 10 items related to the program, alpha = 0.94, mean interitem correlation = 0.59; for the 10 items related to the instructor, alpha = 0.92, mean interitem correlation = 0.56; for the 16 items related to the perceived benefits for students, alpha = 0.97, mean interitem correlation = 0.68; and for the whole Form B with 39 items, alpha = 0.97, mean interitem correlation = 0.51.

### Implementers' Subjective Outcome Evaluation about the S2 Program

Tables 1–4 present the frequency distribution of items of the Subjective Outcome Evaluation Form, including percentages of respondents that evaluated the items at specific ratings, and percentages of respondents with positive responses to each item. Several observations deserve to be highlighted from the findings.

First, the implementers generally had positive perceptions of the S2 program (Table 1), including clear objectives of the teaching units (93.69%), well-planned teaching activities (89.12%), and a strong and sound theoretical support (84.75%). Second, a high proportion of the instructors had a positive evaluation of their own performance in the program implementation (Table 2). For example, 98.60% of the

### TABLE 1
## Summary of the Views of the Program Implementers of the Program

| | 1 | | 2 | | 3 | | 4 | | 5 | | 6 | |
|---|---|---|---|---|---|---|---|---|---|---|---|---|
| | Strongly Disagree | | Disagree | | Slightly Disagree | | Slightly Agree | | Agree | | Strongly Agree | |
| | n | % | n | % | n | % | n | % | n | % | n | % |
| 1. The objectives of the curriculum are very clear. (n = 1,173) | 0 | 0.00 | 12 | 1.02 | 62 | 5.29 | 270 | 23.02 | 764 | 65.13 | 65 | 5.54 |
| 2. The design of the curriculum is very good. (n = 1,169) | 9 | 0.77 | 49 | 4.19 | 138 | 11.80 | 460 | 39.35 | 475 | 40.63 | 38 | 3.25 |
| 3. The activities were carefully planned. (n = 1,167) | 3 | 0.26 | 24 | 2.06 | 100 | 8.57 | 428 | 36.68 | 564 | 48.33 | 48 | 4.11 |
| 4. The classroom atmosphere was very pleasant. (n = 1,171) | 8 | 0.68 | 45 | 3.84 | 142 | 12.13 | 459 | 39.20 | 459 | 39.20 | 58 | 4.95 |
| 5. There was much peer interaction among the students. (n = 1,170) | 2 | 0.17 | 46 | 3.93 | 143 | 12.22 | 496 | 42.39 | 425 | 36.32 | 58 | 4.96 |
| 6. Students participated actively during lessons (including discussions, sharing, games, etc.). (n = 1,161) | 7 | 0.60 | 44 | 3.79 | 165 | 14.21 | 477 | 41.09 | 420 | 36.18 | 48 | 4.13 |
| 7. The program has a strong and sound theoretical support. (n = 1,154) | 6 | 0.52 | 25 | 2.17 | 145 | 12.56 | 420 | 36.40 | 477 | 41.33 | 81 | 7.02 |
| 8. The teaching experience I encountered enhanced my interest in the course. (n = 1,149) | 18 | 1.57 | 66 | 5.74 | 162 | 14.10 | 449 | 39.08 | 406 | 35.34 | 48 | 4.18 |
| 9. Overall speaking, I have very positive evaluation of the program. (n = 1,163) | 17 | 1.46 | 65 | 5.59 | 167 | 14.36 | 468 | 40.24 | 413 | 35.51 | 33 | 2.84 |
| 10. On the whole, students like this curriculum very much. (n = 1,159) | 20 | 1.73 | 64 | 5.52 | 185 | 15.96 | 466 | 40.21 | 401 | 34.60 | 23 | 1.98 |

| | Participants with Positive Responses (Options 4–6) | | Participants with Positive Responses (S2, Experimental Implementation Phase) | | | | | Participants with Positive Responses (S1, Full Implementation Phase) | | | | |
|---|---|---|---|---|---|---|---|---|---|---|---|---|
| | n | % | Total n | n | % | $z^a$ | $p$ | Total n | n | % | $z^b$ | $p$ |
| 1. The objectives of the curriculum are very clear. (n = 1,173) | 1,099 | 93.69 | 344 | 312 | 90.0 | 1.91 | 0.06 | 1,250 | 1,181 | 94.48 | 0.82 | 0.41 |
| 2. The design of the curriculum is very good. (n = 1,169) | 973 | 83.23 | 344 | 252 | 73.26 | **4.14** | 0.00 | 1,247 | 1,008 | 80.83 | 1.53 | 0.13 |
| 3. The activities were carefully planned. (n = 1,167) | 1,040 | 89.12 | 344 | 280 | 81.40 | **3.79** | 0.00 | 1,248 | 1,088 | 87.18 | 1.47 | 0.14 |
| 4. The classroom atmosphere was very pleasant. (n = 1,171) | 976 | 83.35 | 343 | 281 | 81.92 | 0.62 | 0.54 | 1,246 | 1,073 | 86.12 | 1.89 | 0.06 |
| 5. There was much peer interaction among the students. (n = 1,170) | 979 | 83.68 | 344 | 282 | 81.98 | 0.74 | 0.46 | 1,246 | 1,037 | 83.23 | 0.30 | 0.77 |
| 6. Students participated actively during lessons (including discussions, sharing, games, etc.). (n = 1,161) | 945 | 81.40 | 344 | 287 | 83.43 | 0.86 | 0.39 | 1,248 | 1,054 | 84.46 | 1.99 | 0.05 |
| 7. The program has a strong and sound theoretical support. (n = 1,154) | 978 | 84.75 | 343 | 280 | 81.63 | 1.38 | 0.17 | 1,242 | 1,075 | 86.55 | 1.26 | 0.21 |
| 8. The teaching experience I encountered enhanced my interest in the course. (n = 1,149) | 903 | 78.59 | 342 | 255 | 74.56 | 1.57 | 0.12 | 1,244 | 982 | 78.94 | 0.21 | 0.83 |
| 9. Overall speaking, I have very positive evaluation of the program. (n = 1,163) | 914 | 78.59 | 344 | 249 | 72.38 | **2.41** | 0.02 | 1,248 | 952 | 76.28 | 1.35 | 0.18 |
| 10. On the whole, students like this curriculum very much. (n = 1,159) | 890 | 76.79 | 344 | 246 | 71.51 | **2.00** | 0.04 | 1,246 | 976 | 78.33 | 0.91 | 0.37 |

[a] Comparison of implementers' views about the S2 program in the Full Implementation Phase and the Experimental Implementation Phase.

[b] Comparison of implementers' views about the S2 and S1 programs in the Full Implementation Phase.

workers perceived that they were ready to help their students; 98.43% of the workers expressed that they were concerned about the students; 96.76% believed that they had very good professional attitudes. Third, as shown in Table 3, many implementers perceived that the program promoted the development of students, including bonding (88.85%), resilience (85.37%), social competence (91.53%), emotional competence (86.43%), moral competence (90.75%), self-understanding (92.43%), and overall development (91.94%). Fourth, 87.15% of the workers would recommend the program to students with similar needs. Fifth, 79.86%

**TABLE 2**
**Summary of the Views of the Program Implementers about Themselves**

| | 1 Strongly Disagree | | 2 Disagree | | 3 Slightly Disagree | | 4 Slightly Agree | | 5 Agree | | 6 Strongly Agree | |
|---|---|---|---|---|---|---|---|---|---|---|---|---|
| | n | % | n | % | n | % | n | % | n | % | n | % |
| 1. I have a good mastery of the curriculum. (n = 1,155) | 4 | 0.35 | 20 | 1.73 | 124 | 10.74 | 473 | 40.95 | 502 | 43.46 | 32 | 2.77 |
| 2. I prepared well for the lessons. (n = 1,154) | 2 | 0.17 | 16 | 1.39 | 107 | 9.27 | 447 | 38.73 | 533 | 46.19 | 49 | 4.25 |
| 3. My teaching skills were good. (n = 1,133) | 1 | 0.09 | 15 | 1.32 | 107 | 9.44 | 466 | 41.13 | 516 | 45.54 | 28 | 2.47 |
| 4. I have good professional attitudes. (n = 1,141) | 0 | 0.00 | 3 | 0.26 | 34 | 2.98 | 308 | 26.99 | 718 | 62.93 | 78 | 6.84 |
| 5. I was very involved. (n = 1,143) | 4 | 0.35 | 8 | 0.70 | 70 | 6.12 | 327 | 28.61 | 644 | 56.34 | 90 | 7.87 |
| 6. I gained a lot during the course of instruction. (n = 1,143) | 8 | 0.70 | 34 | 2.97 | 150 | 13.12 | 445 | 38.93 | 444 | 38.85 | 62 | 5.42 |
| 7. I cared for the students. (n = 1,144) | 0 | 0.00 | 2 | 0.17 | 16 | 1.40 | 226 | 19.76 | 740 | 64.69 | 160 | 13.99 |
| 8. I was ready to offer help to students when needed. (n = 1,145) | 0 | 0.00 | 3 | 0.26 | 13 | 1.14 | 174 | 15.20 | 752 | 65.68 | 203 | 17.73 |
| 9. I had much interaction with the students. (n = 1,144) | 0 | 0.00 | 14 | 1.22 | 88 | 7.69 | 416 | 36.36 | 552 | 48.25 | 74 | 6.47 |
| 10. Overall speaking, I have very positive evaluation of myself as an instructor. (n = 1,142) | 0 | 0.00 | 10 | 0.88 | 52 | 4.55 | 336 | 29.42 | 693 | 60.68 | 51 | 4.47 |

| | Participants with Positive Responses (Options 4–6) | | Participants with Positive Responses (S2, Experimental Implementation Phase) | | | | | Participants with Positive Responses (S1, Full Implementation Phase) | | | | |
|---|---|---|---|---|---|---|---|---|---|---|---|---|
| | n | % | Total n | n | % | $z^a$ | *p* | Total n | n | % | $z^b$ | *p* |
| 1. I have a good mastery of the curriculum. (n = 1,155) | 1,007 | 87.19 | 339 | 271 | 79.94 | **3.34** | **0.00** | 1,237 | 1,059 | 85.61 | 1.12 | 0.26 |
| 2. I prepared well for the lessons. (n = 1,154) | 1,029 | 89.17 | 330 | 279 | 84.55 | **2.29** | **0.02** | 1,233 | 1,061 | 86.05 | **2.31** | **0.02** |
| 3. My teaching skills were good. (n = 1,133) | 1,010 | 89.14 | 327 | 277 | 84.71 | **2.19** | **0.03** | 1,230 | 1,085 | 88.21 | 0.71 | 0.48 |
| 4. I have good professional attitudes. (n = 1,141) | 1,104 | 96.76 | 338 | 328 | 97.04 | 0.26 | 0.79 | 1,233 | 1,195 | 96.92 | 0.22 | 0.82 |
| 5. I was very involved. (n = 1,143) | 1,061 | 92.83 | 339 | 317 | 93.51 | 0.43 | 0.66 | 1,237 | 1,173 | 94.83 | **2.03** | **0.04** |
| 6. I gained a lot during the course of instruction. (n = 1,143) | 951 | 83.20 | 329 | 266 | 80.85 | 0.99 | 0.32 | 1,234 | 1,027 | 83.23 | 0.02 | 0.99 |
| 7. I cared for the students. (n = 1,144) | 1,126 | 98.43 | 340 | 335 | 98.53 | 0.13 | 0.89 | 1,237 | 1,222 | 98.79 | 0.75 | 0.45 |
| 8. I was ready to offer help to students when needed. (n = 1,145) | 1,129 | 98.60 | 339 | 335 | 98.82 | 0.31 | 0.76 | 1,236 | 1,225 | 99.11 | 1.17 | 0.24 |
| 9. I had much interaction with the students. (n = 1,144) | 1,042 | 91.08 | 337 | 303 | 89.91 | 0.66 | 0.51 | 1,234 | 1,135 | 91.98 | 0.78 | 0.43 |
| 10. Overall speaking, I have very positive evaluation of myself as an instructor. (n = 1,142) | 1,080 | 94.57 | 340 | 320 | 94.12 | 0.32 | 0.75 | 1,236 | 1,187 | 96.04 | 1.69 | 0.09 |

[a,b] See footnotes for Table 1.

of the instructors expressed that they would teach similar programs again in the future. Finally, 83.17% of the respondents indicated that the program helped their own professional development (Table 4)

### Are there any Differences between the Full Implementation Phase and the Experimental Implementation Phase in the Implementers' Evaluation of the S2 Program?

To discover whether implementers' perceptions about the S2 program in the Full Implementation Phase may differ from those in the Experimental Implementation Phase, the percentages of instructors with positive responses to the program in these two stages were compared by employing a series of *z*-tests for two proportions. As can be seen in the Tables 1–4, the percentages of instructors with positive evaluations were basically comparable for the two samples, with no significant differences detected for most of the items

### TABLE 3
## Perceived Effectiveness of the Program by the Program Implementers

| The extent to which the Tier 1 Program (i.e., the program in which all students have joined ) has helped your students: | 1 | | 2 | | 3 | | 4 | | 5 | |
|---|---|---|---|---|---|---|---|---|---|---|
| | Unhelpful | | Not Very Helpful | | Slightly Helpful | | Helpful | | Very Helpful | |
| | n | % | n | % | n | % | n | % | n | % |
| 1. It has strengthened students' bonding with teachers, classmates, and their families. (n = 1,157) | 3 | 0.26 | 126 | 10.89 | 593 | 51.25 | 396 | 34.23 | 39 | 3.37 |
| 2. It has strengthened students' resilience in adverse conditions. (n = 1,155) | 8 | 0.69 | 161 | 13.94 | 606 | 52.47 | 349 | 30.22 | 31 | 2.68 |
| 3. It has enhanced students' social competence. (n = 1,157) | 6 | 0.52 | 92 | 7.95 | 501 | 43.30 | 488 | 42.18 | 70 | 6.05 |
| 4. It has improved students' ability in handling and expressing emotions. (n = 1,157) | 8 | 0.69 | 149 | 12.88 | 523 | 45.20 | 431 | 37.25 | 46 | 3.98 |
| 5. It has enhanced students' cognitive competence. (n = 1,152) | 13 | 1.13 | 164 | 14.24 | 552 | 47.92 | 380 | 32.99 | 43 | 3.73 |
| 6. Students' ability to resist harmful influences has been improved. (n = 1,155) | 13 | 1.13 | 173 | 14.98 | 598 | 51.77 | 335 | 29.00 | 36 | 3.12 |
| 7. It has strengthened students' ability to distinguish between the good and the bad. (n = 1,167) | 7 | 0.60 | 101 | 8.65 | 549 | 47.04 | 465 | 39.85 | 45 | 3.86 |
| 8. It has increased students' competence in making sensible and wise choices. (n = 1,166) | 10 | 0.86 | 141 | 12.09 | 586 | 50.26 | 395 | 33.88 | 34 | 2.92 |
| 9. It has helped students to have life reflections. (n = 1,153) | 12 | 1.04 | 169 | 14.66 | 483 | 41.89 | 428 | 37.12 | 61 | 5.29 |
| 10. It has reinforced students' self-confidence. (n = 1,153) | 13 | 1.13 | 225 | 19.51 | 535 | 46.40 | 343 | 29.75 | 37 | 3.21 |
| 11. It has increased students' self-awareness. (n = 1,163) | 5 | 0.43 | 83 | 7.14 | 541 | 46.52 | 484 | 41.62 | 50 | 4.30 |
| 12. It has helped students to face the future with a positive attitude. (n = 1,165) | 13 | 1.12 | 195 | 16.74 | 547 | 46.95 | 375 | 32.19 | 35 | 3.00 |
| 13. It has helped students to cultivate compassion and care about others. (n = 1,153) | 11 | 0.95 | 153 | 13.27 | 559 | 48.48 | 390 | 33.82 | 40 | 3.47 |
| 14. It has encouraged students to care about the community. (n = 1,154) | 17 | 1.47 | 227 | 19.67 | 545 | 47.23 | 329 | 28.51 | 36 | 3.12 |
| 15. It has promoted students' sense of responsibility in serving the society. (n = 1,151) | 18 | 1.56 | 230 | 19.98 | 562 | 48.83 | 316 | 27.45 | 25 | 2.17 |
| 16. It has enriched the overall development of the students. (n = 1,166) | 6 | 0.51 | 88 | 7.55 | 537 | 46.05 | 479 | 41.08 | 56 | 4.80 |

| | Participants with Positive Responses (Options 3–5) | | Participants with Positive Responses (S2, Experimental Implementation Phase) | | | | | Participants with Positive Responses (S1, Full Implementation Phase) | | | | |
|---|---|---|---|---|---|---|---|---|---|---|---|---|
| | | | | n | % | $z^a$ | p | Total n | n | % | $z^b$ | p |
| 1. It has strengthened students' bonding with teachers, classmates, and their families. (n = 1,157) | 1,028 | 88.85 | 343 | 292 | 85.13 | 1.86 | 0.06 | 1,247 | 1,135 | 91.02 | 1.77 | 0.08 |
| 2. It has strengthened students' resilience in adverse conditions. (n = 1,155) | 986 | 85.37 | 343 | 279 | 81.34 | 1.81 | 0.07 | 1,244 | 1,064 | 85.53 | 0.11 | 0.91 |
| 3. It has enhanced students' social competence. (n = 1,157) | 1,059 | 91.53 | 343 | 313 | 91.25 | 0.16 | 0.87 | 1,246 | 1,163 | 93.34 | 1.68 | 0.09 |
| 4. It has improved students' ability in handling and expressing emotions. (n = 1,157) | 1,000 | 86.43 | 343 | 303 | 88.34 | 0.92 | 0.36 | 1,246 | 1,138 | 91.33 | **3.83** | **0.00** |
| 5. It has enhanced students' cognitive competence. (n = 1,152) | 975 | 84.64 | 343 | 283 | 82.51 | 0.95 | 0.34 | 1,245 | 1,041 | 83.61 | 0.68 | 0.49 |
| 6. Students' ability to resist harmful influences has been improved. (n = 1,155) | 969 | 83.90 | 343 | 268 | 78.13 | **2.47** | **0.01** | 1,245 | 1,026 | 82.41 | 0.97 | 0.33 |
| 7. It has strengthened students' ability to distinguish between the good and the bad. (n = 1,167) | 1,059 | 90.75 | 342 | 309 | 90.35 | 0.22 | 0.83 | 1,247 | 1,121 | 89.90 | 0.71 | 0.48 |
| 8. It has increased students' competence in making sensible and wise choices. (n = 1,166) | 1,015 | 87.05 | 343 | 290 | 84.55 | 1.19 | 0.23 | 1,246 | 1,066 | 85.55 | 1.07 | 0.29 |
| 9. It has helped students to have life reflections. (n = 1,153) | 972 | 84.30 | 343 | 269 | 78.43 | **2.54** | **0.01** | 1,245 | 1,030 | 82.73 | 1.03 | 0.30 |
| 10. It has reinforced students' self-confidence. (n = 1,153) | 915 | 79.36 | 342 | 271 | 79.24 | 0.05 | 0.96 | 1,247 | 1,036 | 83.08 | **2.34** | **0.02** |
| 11. It has increased students' self-awareness. (n = 1,163) | 1,075 | 92.43 | 344 | 320 | 93.02 | 0.37 | 0.71 | 1,244 | 1,173 | 94.29 | 1.83 | 0.07 |
| 12. It has helped students to face the future with a positive attitude. (n = 1,165) | 957 | 82.15 | 343 | 271 | 79.01 | 1.31 | 0.19 | 1,246 | 1,050 | 84.27 | 1.40 | 0.16 |
| 13. It has helped students to cultivate compassion and care about others. (n = 1,153) | 989 | 85.78 | 343 | 268 | 78.13 | **3.39** | **0.00** | 1,243 | 1,054 | 84.79 | 0.68 | 0.50 |
| 14. It has encouraged students to care about the community. (n = 1,154) | 910 | 78.86 | 344 | 251 | 72.97 | **2.30** | **0.02** | 1,243 | 974 | 78.36 | 0.30 | 0.77 |
| 15. It has promoted students' sense of responsibility in serving the society. (n = 1,151) | 903 | 78.45 | 343 | 248 | 72.30 | **2.38** | **0.02** | 1,247 | 957 | 76.74 | 1.00 | 0.32 |
| 16. It has enriched the overall development of the students. (n = 1,166) | 1,072 | 91.94 | 344 | 309 | 89.83 | 1.23 | 0.22 | 1,246 | 1,170 | 93.90 | 1.88 | 0.06 |

[a,b] See footnotes for Table 1.

**TABLE 4**
**Other Aspects of Subjective Outcome Evaluation Based on the Views of the Workers**

If you have a student/client whose needs and conditions are similar to those of your students who have joined the program, will you suggest him/her to participate in this program? (n = 1,152)

| 1 | | 2 | | 3 | | 4 | | Participants with Positive Responses (Options 3–4) | | Participants with Positive Responses (S2, Experimental Implementation Phase) | | | | | Participants with Positive Responses (S1, Full Implementation Phase) | | | | |
|---|---|---|---|---|---|---|---|---|---|---|---|---|---|---|---|---|---|---|---|
| Definitely Will Not Suggest | | Will Not Suggest | | Will Suggest | | Definitely Will Suggest | | | | | | | | | | | | | |
| n | % | n | % | n | % | n | % | n | % | Total n | n | % | $z^a$ | *p* | Total n | n | % | $z^b$ | *p* |
| 14 | 1.22 | 134 | 11.63 | 909 | 78.91 | 95 | 8.25 | 1,004 | 87.15 | 338 | 285 | 84.32 | 1.34 | 0.18 | 1,233 | 1,095 | 88.81 | 1.24 | 0.21 |

If there is a chance, will you teach similar programs again in the future? (n = 1,132)

| 1 | | 2 | | 3 | | 4 | | Participants with Positive Responses (Options 3–4) | | Participants with Positive Responses (S2, Experimental Implementation Phase) | | | | | Participants with Positive Responses (S1, Full Implementation Phase) | | | | |
|---|---|---|---|---|---|---|---|---|---|---|---|---|---|---|---|---|---|---|---|
| Definitely Will Not Teach | | Will Not Teach | | Will Teach | | Definitely Will Teach | | | | | | | | | | | | | |
| n | % | n | % | n | % | n | % | n | % | Total n | n | % | $z^a$ | *p* | Total n | n | % | $z^b$ | *p* |
| 29 | 2.56 | 199 | 17.58 | 811 | 71.64 | 93 | 8.22 | 904 | 79.86 | 335 | 283 | 84.48 | 1.89 | 0.06 | 1,223 | 1,031 | 84.30 | **2.81** | **0.00** |

Do you think the implementation of the program has helped you in your professional growth (e.g., enhancement of your skills)? (n = 1,147)

| 1 | | 2 | | 3 | | 4 | | 5 | | Participants with Positive Responses (Options 3–5) | | Participants with Positive Responses (S2, Experimental Implementation Phase) | | | | | Participants with Positive Responses (S1, Full Implementation Phase) | | | | |
|---|---|---|---|---|---|---|---|---|---|---|---|---|---|---|---|---|---|---|---|---|---|
| Unhelpful | | Not Very Helpful | | Slightly Helpful | | Helpful | | Very Helpful | | | | | | | | | | | | | |
| n | % | n | % | n | % | n | % | n | % | n | % | Total n | n | % | $z^a$ | *p* | Total n | n | % | $z^b$ | *p* |
| 26 | 2.27 | 167 | 14.56 | 514 | 44.81 | 393 | 34.26 | 47 | 4.10 | 954 | 83.17 | 329 | 264 | 80.24 | 1.23 | 0.22 | 1,231 | 1,010 | 82.05 | 0.72 | 0.47 |

[a,b] See footnotes for Table 1.

(28 out of 39 items, 71.79%). Meanwhile, there were a few items over which the percentage of implementers holding positive evaluations increased in the Full Implementation Phase as compared to the Experimental Implementation Phase. For example, while 73.26% of the respondents in the Experimental Implementation Phase agreed that "the design of the curriculum is very good" (Item 2 of Table 1), this figure significantly increased to 83.23% for the present sample ($z = 4.14$, $p < 0.001$). As to the program implementers' views of their own practice, the percentage of respondents who believed that they "have a good mastery of the curriculum" rose from 79.97% in the Experimental Implementation Phase to 87.19% in the Full Implementation Phase ($z = 3.34$, $p < 0.001$). For implementers' perceived effectiveness of the S2 program, 85.78% of the present sample of teachers and social workers considered the program "has helped students to cultivate compassion and care about others", which was higher than the previously reported figure (78.13%) in the Experimental Implementation Phase ($z = 3.39$, $p < 0.001$).

### Are there any Differences between Implementers' Perceptions of S2 and S1 Programs?

To find out whether there are any differences between the implementers' perceptions of the S2 and S1 programs, the percentages of instructors with positive responses toward the two programs were further compared through a set of *z*-tests for two proportions. As can be seen in Tables 1–4, with few exceptions (five out of 39 items, 12.82%), no significant differences between S1 and S2 implementers were identified in the percentages of respondents with positive evaluations. Specifically, a higher percentage of S2 implementers (89.17%) than S1 implementers (86.05%) considered themselves as being prepared well for the lessons ($z = 2.31$, $p < 0.05$). On four items, the proportions of S1 respondents with positive ratings were higher than S2 implementers (i.e., "I was very involved" with 94.83% for S1 and 92.83% for S2; "It has improved students' ability in handling and expressing emotions" with 91.33% for S1 and 86.43% for S2; "It has reinforced students' self-confidence" with 83.08% for S1 and 79.36% for S2; and "will you teach similar program again in the future" 84.30% for S1 and 79.86% for S2).

### Intercorrelations among Different Aspects of Implementers' Subjective Outcome Evaluation

To understand how different aspects of the implementers' perceptions of the S2 program are correlated with each other, three scale scores (views about program, views about one's own performance, perceived program effectiveness) were first calculated by averaging the item scores of each subscale. There are several reasons to support the use of scale scores to represent the respondents' overall perception about the program, the instructors, and the program effectiveness. First, reliability analyses have shown that the three subscales are internally consistent (Cronbach's alpha ranges from 0.92 to 0.97). Second, results of exploratory factor analyses conducted on the three subscales, respectively, also suggest that items of each subscale can be represented by a unifactorial structure (the details of which are not reported here). Besides, to make the scale scores more interpretable, the averaged item score rather than summative score was used and thus the original item rating scale (e.g., 1 to 6 represent strongly disagree to strongly agree) could be retained. After this, means and standard deviations for the three scale scores and item scores on the other three questions (willingness of recommending students to attend the program, willingness of teaching similar programs again, and perceived help in one's own professional growth) were calculated across the 196 schools. Finally, intercorrelations among these variables were computed.

Table 5 shows the means and standard deviations of the six variables and their intercorrelations as well. As can be seen in Table 5, different aspects of program implementers' perceptions about the S2 program were positively correlated. First, respondents' views toward the program were positively associated with the perceptions of their own practice ($r = 0.70$, $p < 0.01$). Second, both the characteristics of program and program implementers were positively correlated with perceived effectiveness of the program (*r*s ranged from 0.67 to 0.71). Third, instructors' views toward the program (program, implementers, and effectiveness) were positively correlated with their willingness to recommend students to join the program (*r*s ranged from 0.55 to 0.66), to teach similar program again (*r*s ranged from 0.58 to 0.63), and with the perceived help in their professional growth (*r*s ranged from 0.61 to 0.66). The findings were statistically significant even after Bonferroni correction.

## DISCUSSION

In this study, subjective outcome evaluation findings based on the perspective of the program implementers (Form B) showed that a high proportion of the respondents had positive perceptions of the S2 program, including the program design, implementers' performance, and program effectiveness. These results thus replicated the earlier findings[12,13,14] and expanded their scope based on a large sample of implementers from 196 schools in the Full Implementation Phase. The present findings were also in line with previous reports based on the views of the students of the same cohort (i.e., students who participated in the S2 program in the Full Implementation Phase), in which it was found that high proportions of the participants evaluated both the program and the instructors positively and regarded the project as beneficial to them[5]. This observation provides support for the effectiveness of the program from a triangulation perspective. Although other findings examining the S2 program of the Full Implementation Phase have yet to be published, similar positive findings were found in process evaluation, interim evaluation, and qualitative

**TABLE 5**
**Means, Standard Deviations, and Intercorrelations of Different Aspects of Implementers' Subjective Outcome Evaluation**

| | Mean | SD | Range | 1 | 2 | 3 | 4 | 5 |
|---|---|---|---|---|---|---|---|---|
| 1. Views towards the course | 4.32 | 0.03 | 1–6 | | | | | |
| 2. Views towards the instructors | 4.63 | 0.02 | 1–6 | 0.70** | 1 | | | |
| 3. Perceived effectiveness | 3.31 | 0.03 | 1–5 | 0.71** | 0.67** | 1 | | |
| 4. Recommend students to join this program | 2.98 | 0.02 | 1–4 | 0.63** | 0.55** | 0.66** | 1 | |
| 5. Teach similar program again | 2.89 | 0.03 | 1–4 | 0.61** | 0.58** | 0.63** | 0.79** | 1 |
| 6. Program has helped instructors in their professional growth | 3.29 | 0.04 | 1–5 | 0.64** | 0.61** | 0.66** | 0.71** | 0.77** |

*Note:* Number of schools = 195; ** $p < 0.01$ * $p < 0.05$.

evaluation of instructors conducted in the Experimental Implementation Phase[21,22,23,24]. Taken together, these research findings consistently demonstrated the successful implementation of the S2 program.

While the above descriptive findings can be interpreted as reflecting the program effectiveness, alternative explanations should not be ignored. Under the "beauty on the beholder side" hypothesis, the workers who were personally involved in program implementation may tend to look at the program effect and their own performance in a more favorable light. Workers are also likely to have cognitive dissonance, which makes it difficult for them to rate the program and themselves in an undesirable manner. The "survival" hypothesis[25] maintains that positive subjective outcome evaluation findings may occur as a result of the workers' anxiety that the program would be cut if the evaluation findings were not positive. However, these alternative explanations could be partially dismissed because negative ratings were also recorded (e.g., whether the workers would teach similar programs again) and the workers responded in an anonymous manner. Nevertheless, following the principle of triangulation, the effectiveness of the Tier 1 Program (S2 level) in the Full Implementation Phase should be further examined by including different sources (e.g., views of school principals and parents) and methods (e.g. prolonged engagement in field studies).

Regarding subjective outcome evaluation differences across programs and program implementation phases, some interesting findings emerge. First, for the perceptions of programs, over 90% of the respondents in all related studies agreed that the objectives of the curriculum are very clear. Such consistent positive findings may be due to the well-designed Tier 1 Program. Aside from the strong theoretical and empirical grounding, a teaching kit has been provided for the Tier 1 Program of each secondary level. In the teaching kit, the curriculum structure, rationales, and unit lesson plans are clearly shown. Together with the professional training courses provided for the program implementers, this would facilitate their understanding of the program rationales and objectives.

Second, regarding the program implementers' perceptions of their own practice, "ready to offer help to students when needed", "cared for students", and "good professional attitudes" were the items with the highest positive responses in all studies. Kumpfer and Alvarado[26] emphasized the close relationship between the characteristics of instructors and the effectiveness of the program. In the Project P.A.T.H.S., the role of implementers is to accompany students as they explore and create their own paths during development. For example, being student oriented and having a high readiness to help students are essential qualities to have in order to foster a positive and caring atmosphere in which students can find it easy to develop maturation and self-exploration. The high correlation ($r = 0.67$) between instructors' views of their own performance and perceived program effectiveness found in the present study offers further support for this important role of implementers.

Third, most of the respondents in the present and previous studies recognized that this program effectively enhanced students' self-awareness, social competence, and overall development. In every unit of the program, there is a 5-min session of self-reflection, which may enable students to review what they have learned and enhance their self-awareness through evaluating their own performance during the lessons, appreciating their success and improving their imperfections. Furthermore, as the activities emphasized in each unit of the Tier 1 Program involve participation, experimentation, reflection, and interaction with peers and instructors, the students' reflective ability, social skills, and overall development could thus be fostered. Of course, further research should be conducted to understand the factors conducive to program effectiveness in the long run.

Despite of the above shared characteristics, it is noteworthy that on a few items of the Subjective Outcome Evaluation Form, the instructors' perceptions about the S1 and S2 programs and their evaluations about the S2 program during the Experimental and Full Implementation Phases were somewhat different. First, for approximately one-fifth of the items, the percentages of implementers with positive evaluations in the Full Implementation Phase were higher than those in the Experimental Implementation Phase, suggesting that instructors in the Full Implementation Phase held more positive views toward the S2 program than in the experimental stage. This finding is consistent with our hypothesis that the program implementation should be better in the Full Implementation Phase than in the Experimental Implementation Phase.

Second, compared to S1 implementers, there was a higher proportion of S2 implementers who perceived themselves as well prepared for the lessons, while a lower proportion of S2 implementers considered themselves as very involved in the program and willing to teach similar programs again. This may be due to the fact that the S2 program was administered 1 year after the S1 program in the same groups of schools. On the one hand, both teachers and social workers implementing the S2 program would be better prepared and more skilled in teaching the course. On the other hand, perhaps the instructors were less than enthusiastic about getting involved with such a program after they had taught the course for 1 year. If this is the case, researchers and instructor trainers need to make more efforts to find strategies for further motivating the instructors; for example, to identify difficulties in the program implementation and provide timely professional help or administrative support.

Echoing findings in the Experimental Implementation Phase[13], significant intercorrelations among different dimensions of instructors' perceptions toward the S2 program were revealed. First, instructors' perceived program features were highly correlated with their own practice. Researchers have always emphasized that the characteristics of program implementers have substantial influences on both the process and outcome of the program implementation[27]. In the Project P.A.T.H.S., it is likely that instructors with good teaching skills, caring for students, and having much interaction with students would create a pleasant classroom atmosphere, enhance students' interests in the lessons, and encourage students' participation. Meanwhile, implementers' perceptions about the program may also influence their performance. Witt and Elliot[27] proposed that the program implementers' practice is guided by their initial judgments about the quality of the program. Taking the Project P.A.T.H.S. as an example, instructors who consider the program as well designed, having clear objectives, and meaningful might be more willing to spend time in preparing the lessons and be more involved. As such, the characteristics of program and program implementers may have mutual effects on each other.

Second, the characteristics of both the program and the implementers were closely related to the perceived program effectiveness, which further supports the key roles of program design and implementers in program success. As noted by Gendreau et al.[28], characteristics of the implementer, program factors, and the environment in which the program is implemented have a critical influence on overall program effects. Donnermeyer and Wurschmidt[29] also pointed out implementers' "level of enthusiasm and support for a prevention curriculum influences their effectiveness because their attitudes are communicated both explicitly and subtly to students during the time it is taught and throughout the remainder of the school day" (pp. 259–260). Several studies[22,30,31] on process evaluation of the Project P.A.T.H.S. have shown high treatment fidelity (>80%), which is believed to be due to the program implementers' positive perception of the program and belief of its effectiveness[32].

Finally, the more positive the instructors evaluated the program, the more likely they would recommend that other students join the program, teach the program again, and perceive the program as useful for their own professional development. In the Project P.A.T.H.S., training courses are provided for instructors before the implementation, and the co-walker scheme is carried out during the program implementation phase. This is important to enhance the workers' understanding towards the program and improve program effectiveness ultimately.

Winefield and Barlow[33] argued that monitoring staff perception was important because "staff have valuable first-hand experience of how, when, and how well programs work" (p. 898). In view of the limited international and local research studies that document the perceptions of workers in youth development or prevention programs, the present study can be regarded as a useful contribution. However, as schools were used as units of analysis, it is important to replicate the present findings using individuals as units of analysis. Despite this limitation, the present study shows that the Tier 1 Program was perceived to be effective by the program implementers and several correlates of subjective outcome evaluation based on the perception of the program implementers were identified.

### ACKNOWLEDGMENTS

The preparation for this paper and the Project P.A.T.H.S. were financially supported by The Hong Kong Jockey Club Charities Trust.
